# Effective gestational weight gain advice to optimize infant birth weight in Japan based on quantile regression analysis

**DOI:** 10.1038/s41598-023-48375-z

**Published:** 2023-11-28

**Authors:** Noriko Sato, Rei Haruyama, Naoyuki Miyasaka

**Affiliations:** 1 Department of Food and Nutrition, Faculty of Human Sciences and Design, Japan Women’s University, 2-8-1 Mejirodai, Bunkyo-ku, Tokyo, 112-8681 Japan; 2 Bureau of International Health Cooperation, National Center for Global Health and Medicine, Tokyo, 162-8655 Japan; 3 Comprehensive Reproductive Medicine, Graduate School of Medical and Dental Sciences, Tokyo Medical and Dental University, Tokyo, 113-8510 Japan

**Keywords:** Predictive medicine, Health care, Disease prevention, Preventive medicine, Reproductive disorders, Medical research, Epidemiology

## Abstract

The optimal range of gestational weight gain (GWG) was recently raised in Japan. This may help reduce small-for-gestational-age (SGA) infants, but may also increase large-for-gestational-age (LGA) infants. This study performed hypothetical experiments to determine effective GWG advice based on quantile regression analysis. In a total of 354,401 singleton pregnancies registered in the perinatal database of the Japan Society of Obstetrics and Gynecology (2013–2017), the proportions of SGA and LGA were 9.33% and 11.13%, respectively. Using regression coefficients of GWG across the birth weight-for-gestational-age quantile distribution, we analyzed changes in their proportions by simulating a uniform 3-kg extra increase in GWG or an increase or decrease based on GWG adequacy. A hypothetical experiment of a uniform increase in GWG resulted in SGA and LGA proportions of 7.26% (95% confidence interval 7.15–7.36) and 14.51% (14.37–14.66), respectively. By contrast, assuming a 3-kg increase in women with inadequate GWG and a 3-kg decrease in women with excessive GWG resulted in SGA and LGA proportions of 8.42% (8.31–8.54) and 11.50% (11.37–11.62), respectively. Our real-world data analysis suggests that careful adjustment of GWG based on GWG adequacy will be effective in optimizing infant birth weight in Japan

## Introduction

Gestational weight gain (GWG) is a potentially modifiable factor that can prevent the occurrence of adverse maternal and infant outcomes, including small-for-gestational-age (SGA) and large-for-gestational-age (LGA)^1, 2^. Japan is a country with one of the lowest birth weights in the world^3^. One of the reasons for this is thought to be the high prevalence of women classified as underweight and strict weight management of GWG^3–5^. As a result, in 2021, the Japanese Society of Obstetrics and Gynecology (JSOG) raised the GWG target levels. The guidance now advises women classified as underweight (i.e., prepregnancy body mass index [BMI] < 18.5 kg/m^2^) to gain 12–15 kg (versus 9–12 kg previously), normal weight (i.e., 18.5 ≤ BMI < 25) to gain 10–13 kg (versus 7–12 kg), overweight (i.e., 25 ≤ BMI < 30) to gain 7–10 kg (versus no official recommendation), and obese (i.e., BMI ≥ 30) to gain ≤ 5 kg (versus no official recommendation) by 40 weeks of gestation. In other words, the lower limit of the target level was uniformly increased by 3 kg for normal weight and underweight women, who are the majority in Japan. Shortly after the new guidance was issued, GWG growth charts containing both the upper and lower limits of GWG by gestational age were recently developed^6^. However, the effectiveness of using these charts has not yet been verified.

Generally, GWG advice is given according to prepregnancy BMI. However, recent studies suggest that the effect size of GWG on birth weight varies greatly by birth-weight quantiles; the impact of GWG on birth weight is much larger in the 90th than in the 10th birth-weight percentile^7, 8^. Therefore, a uniform extra 3-kg increase in women according to their prepregnancy BMI could lead to an excessive proportion of LGA infants. It would be useful in real-life clinical practice to know how the estimated proportions of SGA and LGA would change when GWG advice is given based solely on prepregnancy BMI and when it is adjusted based on GWG adequacy using the GWG growth charts.

Given this background, the objectives of the present study were, first, to examine the effect of GWG at different percentiles of birth-weight distribution by quantile regression analysis using a nationwide perinatal database with a large sample size, and second, to perform hypothetical experiments to compare the proportions of SGA and LGA infants between the strategy of uniformly increasing GWG according to prepregnancy BMI and the strategy of adjusting GWG based on GWG adequacy.

## Results

### Population characteristics

Table 1 shows the population characteristics. Table S1 shows the classifications according to GWG adequacy by prepregnancy BMI category. Approximately 20% of the women were underweight while 7% and 1% of the women were overweight and obese, respectively. Approximately 50% showed inadequate GWG, whereas 23% showed excessive GWG.

**Table 1:** Characteristics of study population derived from the Japan Society of Obstetrics and Gynecology Perinatal Database, 2013–2017 (total number = 354,401).

	Median (IQR), mean [SD] or n (%)			
	Total	SGA (n = 33,102)	AGA (n = 281,843)	LGA (n = 39,456)
Prepregnancy BMI (kg/m^2^)	20.4 (18.8, 22.1)	19.9 (18.5, 21.7)	20.2 (18.8, 22.0)	21.0 (19.5, 23.1)
GWG (kg/40 weeks)	10.4 [3.9]	9.4 [3.8]	10.3 [3.8]	11.6 [4.1]
Height (cm)	158.3 [5.4]	156.7 [5.4]	158.3 [5.4]	159.9 [5.4]
Age (year)	31.4 [5.7]	31.3 [5.7]	31.4 [5.7]	31.9 [5.8]
Fetal sex (male)	184,040 (51.9)	16,964 (51.2)	145,695 (51.7)	21,381 (54.2)
ART	60,850 (17.2)	5113 (15.4)	47,536 (16.9)	8201 (20.8)
Smoking	54,055 (15.3)	2484 (7.5)	18,277 (6.5)	2268 (5.7)
HDP	23,029 (6.5)	6060 (18.3)	16,480 (5.8)	1796 (4.6)
DM	24,336 (6.9)	1660 (5.0)	15,153 (5.4)	3357 (8.5)
Autoimmune disease	20,170 (5.7)	498 (1.5)	2634 (0.9)	243 (0.6)

*SD* standard deviation, *IQR* interquartile range, *SGA* small for gestational age, *AGA* appropriate for gestational age, *LGA* large for gestational age, *BMI* body mass index, *GWG* gestational weight gain, *ART* assisted reproductive technology, *HDP* hypertensive disorders of pregnancy, *DM* diabetes mellitus.

### Unequal effects of GWG on BWGA across quantiles

**Figure 1:**
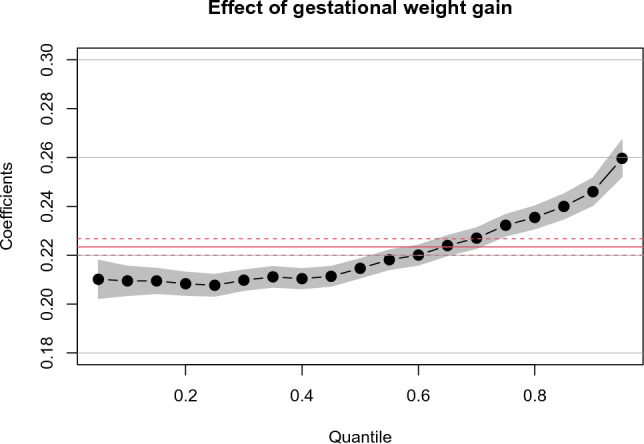
Effect of gestational weight gain (GWG) on birth weight for gestational age (BWGA) across BWGA quantiles. The black dots and gray bands indicate the coefficient estimates at each quantile and 95% confidence interval (CI), respectively, which were obtained using multivariate quantile regression analysis. The red solid and dashed lines indicate the OLS coefficients (0.223) and their 95% CIs, respectively, which were obtained using conventional multivariable linear regression analysis. The covariates were maternal age, height, prepregnancy BMI, smoking status, hypertensive disorders of pregnancy, diabetes mellitus, autoimmune disease, and assisted reproductive technology. The coefficients refer to a change in outcome (BWGA) in response to a change of 1 standard deviation equivalent of GWG.

Figure 1 shows the coefficient estimates for GWG on birth weight for gestational age (BWGA) across quantiles obtained by multivariable quantile regression analysis. The coefficient estimate at the 50th quantile (0.215) was close to that of the ordinary least squares (0.223), but it was small (0.210) in the smaller quantile and large (0.246) at the 90th quantile. As a reference, Tables S2 and S3 show the results of the conventional univariable and multivariable linear regression analyses. The coefficient estimates for maternal factors across all the quantiles derived from multiple quantile regression analysis are also included in Table S3, showing that all factors were associated with BWGA but their effect size varied depending on the quantiles.

A sensitivity analysis produced similar results and confirmed that unequal effects of GWG on BGWA could be detected even in the population excluding high-risk pregnancies (Fig. S2).

### Hypothetical experiment to investigate the effects of uniform GWG increases on the proportions of SGA and LGA

In the present study population, the proportions (%) of SGA and LGA were 9.33 and 11.13, respectively (Table 2a). Among multiple factors, GWG is a modifiable factor that can reduce the proportions of SGA and LGA. Following general guidance, BWGA Z-scores were predicted using a quantile regression model, experiment 1 assumed a uniform 3-kg increase in GWG at 40 weeks for underweight and normal weight subjects. This hypothetical experiment 1 led to a 2.07% decrease in SGA, but a 3.38% increase in LGA (Table 2b).

**Table 2:** Effects of different hypothetical strategies on the proportions of SGA and LGA.

	SGA (%)	LGA (%)
(a) Observed data (current status)		
Underweight	2.33	1.32
Normal weight	6.35	8.43
Overweight/obese	0.65	1.38
Total	9.33	11.13
(b) Estimates in hypothetical experiment 1		
Underweight	1.76 (1.70–1.81)	1.89 (1.83–1.95)
Normal weight	4.85 (4.76–4.93)	11.24 (11.11–11.37)
Overweight/obese	0.65 (0.62–0.68)	1.38 (1.34–1.43)
Total	7.26 (7.15–7.36)	14.51 (14.37–14.66)
(c) Estimates in hypothetical experiment 2		
Underweight	1.89 (1.83–1.94)	1.62 (1.56–1.66)
Normal weight	5.53 (5.43–5.62)	9.43 (9.30–9.55)
Overweight/obese	0.61 (0.58–0.64)	1.47 (1.42–1.52)
Total	8.02 (7.91–8.13)	12.51 (12.37–12.64)
(d) Estimates in hypothetical experiment 3		
Underweight	2.39 (2.33–2.46)	1.23 (1.18–1.27)
Normal weight	6.67 (6.57–6.77)	7.62 (7.51–7.73)
Overweight/obese	0.68 (0.65–0.72)	1.27 (1.23–1.32)
Total	9.74 (9.62–9.86)	10.12 (10.00–10.24)
(e) Estimates in hypothetical experiment 4		
Underweight	1.94 (1.88–2.00)	1.53 (1.48–1.57)
Normal weight	5.84 (5.75–5.94)	8.61 (8.50–8.72)
Overweight/obese	0.64 (0.61–0.67)	1.36 (1.31–1.41)
Total	8.42 (8.31–8.54)	11.50 (11.37–11.62)

Hypothetical experiment 1: uniform 3-kg GWG increase in underweight and normal weight women.

Hypothetical experiment 2: only a 3-kg GWG increase in underweight, normal weight, and overweight women with inadequate GWG.

Hypothetical experiment 3: only a 3-kg GWG decrease in underweight, normal weight, and overweight women with excessive GWG.

Hypothetical experiment 4: a 3-kg GWG increase in underweight, normal weight, and overweight women with inadequate GWG and a 3-kg GWG decrease in underweight, normal weight, and overweight women with excessive GWG.

### Hypothetical experiments to investigate the effects of GWG changes based on GWG adequacy on the proportions of SGA and LGA

**Figure 2:**
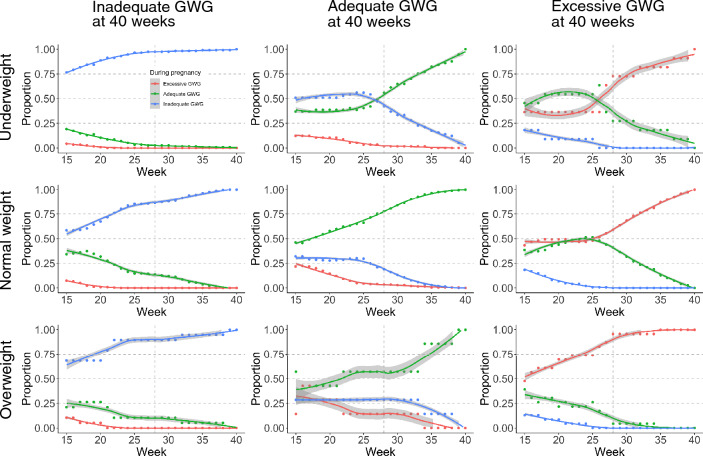
Concordance of gestational weight gain (GWG) adequacy across gestational weeks. Proportion of GWG adequacy at each week of pregnancy among groups classified by GWG adequacy at 40 weeks. The solid line and gray band indicate the regression line and 95% confidence interval. The top, middle, and bottom panels indicate underweight, normal weight, and overweight women, respectively, and the left, middle, and right panels indicate inadequate, adequate, and excessive GWG at 40 weeks, respectively. The number and percentage of subjects comprising each panel are shown in Table S5. The panels on the left show that > 70% of women with inadequate GWG at 40 weeks showed inadequate GWG from mid-gestation. The panels on the right show that most women with excessive GWG at 40 weeks showed excessive GWG from around 28 weeks of gestation.

A hypothetical experiment to examine the increase or decrease in GWG based on GWG adequacy presumes that GWG adequacy does not change much over gestational weeks. Therefore, we verified the concordance of GWG adequacy from 15 to 40 weeks and that at 40 weeks using gestational weight data of subpopulations (Fig. 2). The results revealed that many (> 70%) of those with inadequate GWG at 40 weeks had inadequate GWG from 15 weeks of gestation. In addition, more than half of the women who had excessive GWG at 40 weeks showed excessive GWG from at least 28 weeks of gestation.

Then, the following three hypothetical experiments were conducted: one involving a 3-kg GWG increase in women with inadequate GWG (hypothetical experiment 2); one involving a 3-kg GWG decrease in women with excessive GWG (hypothetical experiment 3); and one involving a 3-kg GWG increase in women with inadequate GWG and a 3-kg GWG decrease in women with excessive GWG (hypothetical experiment 4) (Table 2c–e). While the hypothetical experiments 2 and 3 resulted in 1.38% increase in LGA and undesired increase in SGA, respectively, the hypothetical experiment 4 resulted in a 0.91% decrease in SGA and only a 0.37% increase in LGA (Table 2c–e).

## Discussion

Based on the results of the quantile regression models using a large nationwide perinatal database in Japan, we investigated what strategy regarding GWG would be effective in decreasing SGA while not substantially increasing LGA. The results revealed that the strategy of uniformly increasing GWG according to prepregnancy BMI decreased SGA by 2.1%, but increased LGA by 3.4%, more than 1.5 times the rate. By contrast, the strategy of increasing GWG in the inadequate group and decreasing GWG in the excessive group reduced SGA while only increasing LGA by less than half that rate. Sub-analyses were also performed, and showed that GWG adequacy remained concordant through mid-to-late gestation in most women.

GWG has unequal effects on birth weight by quantile. A previous study that conducted a quantile regression analysis reported that an uniform reduction in GWG was effective for reducing the risk of macrosomia^7^. This strategy may be effective in countries plagued with obesity. By contrast, in Japan, low birth weight is more problematic, as it is characterized by a high proportion of lean women and a significantly lower GWG compared with other countries^3, 6^. While the optimal GWG range according to BMI was recently raised to address this issue, the present results suggest that a uniform 3-kg GWG increase among underweight and normal weight women may unintentionally increase the proportion of LGA by 3.4%. This large increment was not surprising because the GWG regression coefficient was abruptly elevated in the higher BWGA quantile (Fig. 1). This finding supports the importance of recognizing the unequal effects of GWG across birth-weight quantile distributions.

For GWG management in clinical practice, it is important to assess whether weight gain is within the adequate range throughout pregnancy. Using the recently developed GWG charts, the present study first analyzed whether the adequacy of GWG is concordant through 15–40 weeks of gestation (Fig. 2). The results showed that 70% of women with inadequate GWG at 40 weeks had already been assessed as inadequate as early as 20 weeks, regardless of prepregnancy BMI. More than 60% of women with excessive GWG at 40 weeks were concordantly assessed as excessive at 30 weeks. It has been reported that weight gain in mid-to-late gestation is associated with an increased risk of low birth weight and/or macrosomia^1^. Therefore, it was considered reasonable to conduct hypothetical experiments to evaluate the strategies in which GWG increased or decreased according to GWG adequacy.

In the hypothetical experiments, the effects of 3-kg increases on BWGA in the case of inadequate GWG and 3-kg decreases in the case of excessive GWG were compared, both alone and in combination (Table 2). To the best of our knowledge, no such approach leveraging the latest GWG charts has been reported. The value of 3 kg is not important because it may be difficult to change the weight by 3 kg. More importantly, due to the inequality of the GWG effect, simply advising women to increase their GWG will inevitably lead to an increase in LGA. The present study clearly demonstrated the importance of recommending both an increase in GWG to women with inadequate GWG and a decrease in GWG to women with excessive GWG.

The main strength of the present study is the large study size, which comprised nationwide data representing general Japanese population. The large-scale database with detailed information about maternal and neonatal characteristics made it possible to estimate the effects of multiple maternal factors associated with birth weight^7, 9–16^, including GWG, at various BWGA distribution quantiles (Table S3). The second strength of this study is that it was performed shortly after new GWG guidelines were published. Dissemination of the present results might help prevent clinicians from focusing only on the increased GWG target. An increase in LGA may be unintentionally brought if attention is paid only to the increase in GWG according to pre-pregnancy BMI, adhering to the new guidance**.** Third, we used a unique approach of evaluating the effect of hypothetical strategies. The advantage of taking such an approach is that it allows us to judge whether a strategy is good or bad without having to conduct more difficult large-scale trials in real life.

However, this study also has a few limitations. Because the amount of weight gain was increased by 3 kg in the new Japanese guidance, the hypothetical weight change in the intervention was analyzed as 3 kg, but there was no specific rationale for this value. In reality, it may be difficult to manage a 3 kg weight gain or loss throughout the entire gestation period. However, even if the 3-kg value itself were different, the unequal GWG effect across birth-weight distributions would still be the same; therefore, we nevertheless conclude that focusing solely on raising GWG would not ultimately change the trend of increasing LGA. Further research is therefore necessary to verify how the SGA and LGA rates will change as a result of the weight control in the new guidelines. In addition, although we took advantage of a large-scale perinatal database and included multiples factors associated with birth weight in our analysis, data on other related factors (e.g. socioeconomic status) were not available from the database and therefore not included.

## Conclusion

Although raising the GWG target was an important step for optimizing birth weight in Japan, it is not sufficient. Our quantile regression analysis suggest that assessing GWG adequacy using the newly developed GWG standard charts and recommending weight gain when it is inadequate and weight loss when it is excessive may be effective in optimizing infant birth weight.

## Methods

### Ethics

This study was approved by the Research Ethics Review Committee at Tokyo Medical and Dental University (No. M2019-226, 2019/11/22) and the Clinical Research Management and Review Committee of the Japan Society of Obstetrics and Gynecology (No. 100, 2020/7/27). All methods were performed in accordance with the relevant guidelines and regulations of the institutions. Informed consent was obtained from patients for the use of their data, which were collected during routine clinical practice for medical research purposes.

### Data source and study population

We used data from January 2013 to December 2017 of the JSOG perinatal database^17^. This database consists of a nationwide registry that contains clinical information on all births after 22 weeks of gestation at registered participating obstetric facilities. Over the study period, a total of 1,128,073 births, including 6654 stillbirths, were anonymously recorded in the database, corresponding to 22.7% of all births in Japan published as national statistics^18^.

Figure S1 shows the study design. First, singleton pregnancies (91.3% of the initial data) were selected by excluding multiple pregnancies, stillbirths, cases with congenital malformation, and maternal deaths. Pregnancies with a gestational age < 28 or > 41 weeks were excluded, since the formula for GWG (kg/40 weeks) estimation used in this study was deduced from data that excluded extremely preterm births (< 28 weeks)^8^ and the GWG charts for assessing GWG adequacy was set up to 41 weeks^6^. Multiparas were excluded to avoid counting the same women during the study period of 5 years. Subjects were also excluded when any of the following data were missing or implausible: maternal age, parity, prepregnancy weight, weight upon delivery, height, gestational age, smoking status, use of ART, infant sex, or birth weight. Maternal height < 50 cm or > 272 cm and maternal weight < 3 kg or > 200 kg were considered implausible. Subsequently, women with outlying height data (values outside ± 3 standard deviations [SD], equivalent to > 175.7 or < 141 cm), and weight data (outside ± 3 SD, equivalent to > 81.3 or < 24.7 kg) were excluded. 97.5% of the subjects excluded with missing or implausible values were women whose weight and/or height were missing or outliers. Between the excluded and the included participants, there was little or no difference in birth weight, gestational age, infant sex or maternal age. The difference in birth weight (the mean [SD] of the excluded vs. the included was 2873 [537] and 2901 [463], respectively) was 28 g. The difference in gestational age (the median (IQR) of the excluded vs. the included were 39.14 (38.00, 40.14) and 39.29 (38.14, 40.14), respectively) was 0.15 weeks. There was no difference between the excluded and included in the male proportion (51.9%) and maternal age (31.4 years). Finally, a total of 354,401 primipara singleton pregnancies (31.4% of initial data) were eligible for analysis. For the sensitivity analysis with lower-risk pregnancies, 178,199 pregnancies (15.8% of the initial data) were further selected by excluding women with HDP, DM, autoimmune disease, ART, and smoking during pregnancy and those under 20 or over 35 years old.

### Gestational weight gain at 40 weeks

GWG was calculated as the difference between maternal weight at 40 weeks of gestation and prepregnancy weight. In other cases when the mother did not deliver at 40 weeks, a predicted value of GWG at 40 weeks was calculated from the rate of weight gain as described previously^8, 19^. The rate of weight gain was calculated for each woman based on a simple linear regression model for the relationship between gestational week and gestational weight.

### Adequacy of GWG by prepregnancy BMI

The recommended GWG ranges at 40 weeks are 12–15, 10–13, 7–10, and ≤ 5 kg for women classified as underweight, normal weight, overweight, and obese, respectively^6^. The recommended GWG ranges at each week of gestation for each BMI category were based on the GWG growth charts published by Morisaki et al.^6^. We classified the subjects whose GWG was within the appropriate range specific to the corresponding BMI as adequate, below the range as inadequate, and above the range as excessive. This classification was not applied to the obese group because no lower limit was set.

### Concordance of GWG adequacy over the gestational period

In clinical practice, guidance on weight gain during gestation is often provided in the second trimester; however, it is unclear to what extent the GWG adequacy assessed before 40 weeks using GWG growth charts is concordant with the GWG adequacy determined at 40 weeks. To overcome this challenge, we investigated the concordance of GWG adequacy from 15 to 40 weeks using weight gain data over the gestational period from a subsample of 859 women in a single-center pregnancy cohort^8^. The details are described in the supporting information.

### Outcome measure

The birth weight-for-gestational-age (BWGA) Z-scores and percentiles were calculated using Japanese neonatal anthropometric charts, which are specific to gestational age, infant sex, and parity^20^. SGA and LGA infants were defined as having a BWGA below the 10th and above the 90th percentile, respectively.

### Statistical analysis

Descriptive statistics for the variables were presented as the mean [SD] or median and interquartile range for continuous variables, and n and percentage for categorical variables.

Multivariate quantile regression was used to analyze the effect of GWG on BWGA Z-scores at different quantiles of the distribution. The covariates were continuous variables such as maternal age, height, prepregnancy BMI (log-transformed because of the skewness of the BMI distribution), and dichotomous categorical variables such as smoking status during pregnancy, HDP, DM, autoimmune disease, and ART. In general, maternal age, height, BMI, and smoking are adjusted as covariates for the association study between birth weight and gestational weight gain^7, 11^. In addition, we added HDP, DM, autoimmune disease and ART as covariates since their significant association with birth weight were found in the large-scale JSOG perinatal database (Table S2). The model that incorporated these covariates showed the smallest Akaike’s Information Criterion (AIC) scores across all the quantiles, indicating that its fitting was the best (Table S4). Continuous variables were scaled to have a zero mean and unit variance by Z-score transformation to compare effect sizes across the investigated characteristics. Dichotomous categorical variables were coded as 0 (absence) or 1 (presence). The potential modifying effect of BMI on the GWG-BWGA association was accounted for by including the interaction of BMI and GWG in the model. Classical ordinary least squares linear regression models were also fitted with the same variables for comparison.

We estimated the effects of hypothetical GWG change experiments using coefficient estimates from the multivariate quantile regression models. A detailed description of the quantile-based calculations is available in Supporting Methods 2. We calculated 95% confidence intervals with the bootstrap method (iterations of bootstrap resampling = 2000).

R software (version 4.2.3) was used for the statistical analyses. Quantile regression models were fitted using the quantreg R package (version 5.95)^21^.

### Supplementary Information

Supplementary Information.

## Data Availability

The perinatal databases used in this study are available from the Japan Society of Obstetrics and Gynecology (JSOG), but access to these data is limited and not publicly available. However, data are available from the corresponding author (NS) upon reasonable request and with permission from the JSOG.
