# Supplementary material for: Effective gestational weight gain advice to optimize infant birth weight in Japan based on quantile regression analysis
Source: Sci Rep. 2023 Nov 28;13:20954. doi: 10.1038/s41598-023-48375-z (PMC10684669; doi:10.1038/s41598-023-48375-z)
Supplement: Supplementary file 1 — Supplementary Information. [file 41598_2023_48375_MOESM1_ESM.pdf]

Supplementary information

Gestational weight gain advice to optimize infant birth weight in Japan: A quantile regression analysis using a nationwide perinatal database

Noriko Sato, MD PhD; Rei Haruyama, MD MSc; Naoyuki Miyasaka MD PhD

Page		
2	Supporting method1	Concordance of GWG adequacy over the gestational period
3	Supporting method2	Calculation of BWGA Z-scores after hypothetical experiments
4	Figure S1	Flow diagram of the study population
5	Figure S2	Sensitivity analysis of multivariable quantile regression model
6	Table S1	Classification according to gestational weight gain adequacy at 40 weeks in the study population derived from JSOG Database (n = 354,401)
7	Table S2	Univariable linear regression analysis (OLS method)
8	Table S3	Multivariable linear regression (OLS method) and quantile regression analyses
9	Table S4	Akaike's Information Criterion (AIC) scores of the multivariable quantile regression models
	Table S5	Characteristics of a subsample of 859 women
10	Table S6	Classification according to gestational weight gain adequacy at 40 weeks in the subsample population (n = 859)

Supporting Method 1.

Concordance of GWG adequacy over the gestational period

We performed a retrospective cohort analysis of pregnant women who delivered their newborns at Tokyo Medical and Dental University Hospital from 2013 to 2017 (1). Maternal weight measurements during gestation were performed 11.3 ± 3.1 times for each woman. For consistency with the conditions of the main analysis, included were women with a primipara singleton delivery. As a result, 859 women were eligible (Table S5). All data analyzed had a good fit in the simple linear regression for the relationship between gestational weeks and weight gain, so GWG at each gestational week, including at 40 weeks, was calculated from the rate of weight gain similar to the main analysis.

First, the women were classified as underweight, normal weight, overweight, or obese based on their prepregnancy BMI. GWG adequacy at 40 weeks was determined based on JSOG guidance (the adequate range for GWG at 40 weeks was 12–15, 10–13, and 7–10 kg for underweight, normal weight, and overweight, respectively) (2). Then, underweight, normal weight, and overweight women were further divided into the following three groups, resulting in nine groups (Table S6): inadequate, adequate, and excessive GWG. Next, GWG adequacy at each week of gestation was determined based on the GWG growth chart (3). In each of the nine groups, the proportion of GWG adequacy classifications was calculated from 15 to 40 weeks. A graph was drawn with the x-axis representing the weeks of gestation and the y-axis representing the proportion to show, for example, the result that the proportion of those with inadequate GWG exceeded 75% from 15 weeks throughout gestation in the underweight group with inadequate GWG at 40 weeks (Figure 2).

Supporting Method 2.

Calculation of BWGA Z-scores after hypothetical experiments

We estimated the effects of hypothetical GWG change experiments using coefficient estimates from the quantile regression models in a similar manner to that previously described (4).

We calculated coefficient estimates for all variables in the tauth conditional quantiles in the quantile regression analysis. The estimated conditional quantiles were 5th, 10th, 15th, 20th, 25th, 30th, 35th ,40th, 45th, 50th, 55th, 60th, 65th, 70th, 75th, 80th, 85th, 90th, and 95th. Each infant was allocated into 19 parts across conditional quantiles.

In the hypothetical experiments, GWG parameters were changed. Therefore, the resultant outcome, BWGA Z-score (post), was expressed as:

$$BWGA\ Z-score\ (post) = BWGA\ Z-score\ (pre) + GWG\ change \times \left(Coef_{GWG_i} + Coef_{GWGXBMI_i} \times BMI\right)$$

where BWGA Z-score (post) is the BWGA Z-score after a given hypothetical experiment, BWGA Z-score (pre) is the original BWGA Z-score, GWG change is the normalized value of GWG change in the hypothetical experiment, $Coef_{GWG\ i}$ and $Coef_{GWGx\ BMI\ i}$ are coefficient estimates of GWG and GWG × body mass index (BMI) from the quantile regression at the location of BWGA Z-score in the *i*th range, and BMI is the normalized log-transformed prepregnancy BMI value.

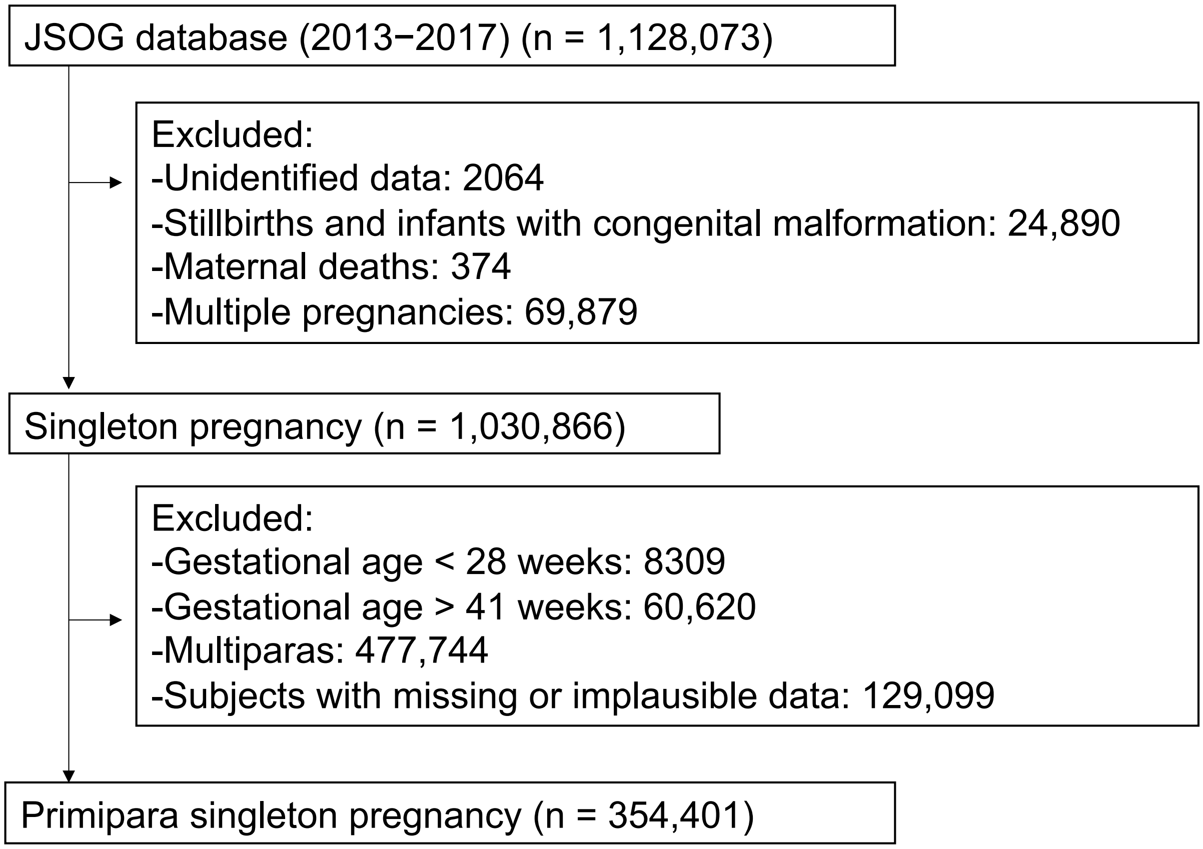

FIGURE S1 Flow diagram of the study population. The Japan Society of Obstetrics and Gynecology (JSOG) Perinatal Database from January 2013 to December 2017 was used in this study.

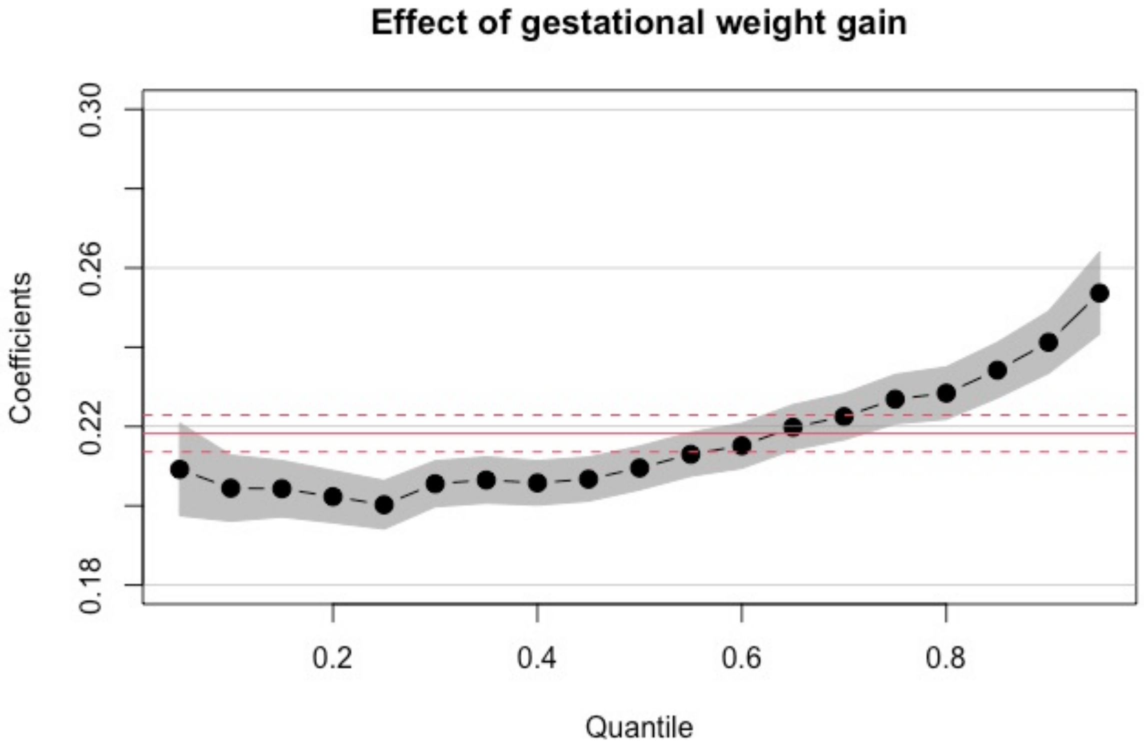

FIGURE S2. Sensitivity analysis

Multivariable quantile regression analysis, excluding the women with hypertensive disorders of pregnancy, diabetes mellitus, autoimmune disease, assisted reproductive technology, smoking during pregnancy, under 20 and over 35 years old were performed. The black dots and gray bands indicate the coefficient estimates at each quantile and 95% confidence interval (CI), respectively, which were obtained using multivariate quantile regression analysis. The red solid and dashed lines indicate the OLS coefficients (0.218) and their 95% CIs (0.214, 0.223), respectively, which were obtained using conventional multivariable linear regression analysis. The covariates were maternal age, height, prepregnancy BMI.

TABLE S1. Classification according to gestational weight gain adequacy at 40 weeks in the study population derived from the Japan Society of Obstetrics and Gynecology Perinatal Database, 2013−2017 (n = 354,401)

		**GWG adequacy (at 40 weeks)**				
		**Inadequate**	**Adequate**	**Excessive**	**NA**	Total
**Underweight**	n	45,297	15,226	7522	0	68,045
	(%)	(12.78%)	(4.3%)	(2.12%)	(0%)	(19.2%)
**Normal weight**	n	113,980	81,803	62,480	0	258,263
	(%)	(32.16%)	(23.08%)	(17.63%)	(0%)	(72.87%)
**Overweight**	n	9511	6290	8713	0	24,514
	(%)	(2.68%)	(1.77%)	(2.46%)	(0%)	(6.91%)
**Obese**	n	0	0	2036	1543	3579
	(%)	(0%)	(0%)	(0.57%)	(0.44%)	(1.01%)
**Total**	n	168,788	103,319	80,751	1543	354,401
	(%)	(47.62%)	(29.15%)	(22.78%)	(0.44%)	(100%)

NA: not applicable.

TABLE S2. Univariable linear regression analysis (Ordinary least squares regression method) for maternal factors on birthweight for gestational age (BWGA) Z-score

Variables	Coefficients	95% confidence interval	*p*-Value
Gestational weight gain (normalized)	0.176	0.173-0.179	< 2e-16
Hypertensive Disorders of Pregnancy	-0.533	-0.546 to -0.520	< 2e-16
Autoimmune disease	-0.271	-0.307 to -0.236	< 2e-16
Prepregnancy BMI (log & normalized)	0.133	0.129-0.136	< 2e-16
Diabetes mellitus	0.189	0.175-0.204	< 2e-16
Height (normalized)	0.178	0.174-0.181	< 2e-16
Assisted Reproductive Technology	0.114	0.105-0.123	< 2e-16
Smoking	-0.082	-0.096 to -0.068	< 2e-16
Age (normalized)	0.034	0.031-0.038	< 2e-16

BMI, body mass index

TABLE S3. Multivariable linear regression (Ordinary least squares regression method) and quantile regression analyses for maternal factors on birthweight for gestational age (BWGA) Z-score

Variables	Upper row, coefficients; Lower row, 95% confidence intervals*							
	Linear regression (OLS)	Quantile regression						
		Quantiles						
		**5th**	**10th**	**25th**	**50th**	**75th**	**90th**	**95th**
(Intercept)	0.072	-1.491	-1.109	-0.53	0.08	0.691	1.261	1.611
	0.068, 0.076	-1.501, -1.482	-1.116, -1.102	-0.535, -0.525	0.075, 0.084	0.686, 0.696	1.255, 1.267	1.602, 1.62
Gestational weight gain (normalized)	0.223	0.210	0.210	0.208	0.215	0.232	0.246	0.260
	0.220, 0.227	0.202, 0.218	0.204, 0.215	0.203, 0.212	0.211, 0.219	0.228, 0.237	0.24, 0.252	0.252, 0.267
Hypertensive Disorders of Pregnancy	-0.653	-1.004	-0.977	-0.831	-0.626	-0.491	-0.421	-0.393
	-0.666, -0.641	-1.033, -0.976	-1.003, -0.951	-0.853, -0.809	-0.645, -0.607	-0.509, -0.473	-0.446, -0.395	-0.422, -0.365
Autoimmune disease	-0.222	-0.262	-0.264	-0.249	-0.220	-0.216	-0.194	-0.154
	-0.256, -0.189	-0.338, -0.185	-0.348, -0.179	-0.3, -0.197	-0.263, -0.178	-0.261, -0.172	-0.27, -0.119	-0.242, -0.067
Prepregnancy BMI (log & normalized)	0.187	0.136	0.149	0.166	0.185	0.207	0.233	0.247
	0.184, 0.19	0.128, 0.144	0.143, 0.155	0.161, 0.17	0.181, 0.189	0.202, 0.212	0.227, 0.239	0.239, 0.255
Diabetes mellitus	0.184	0.134	0.115	0.116	0.140	0.205	0.270	0.362
	0.170, 0.198	0.098, 0.17	0.091, 0.138	0.097, 0.135	0.122, 0.157	0.186, 0.224	0.241, 0.298	0.325, 0.398
Height (normalized)	0.175	0.162	0.164	0.173	0.175	0.174	0.185	0.187
	0.171, 0.178	0.155, 0.17	0.158, 0.17	0.168, 0.177	0.171, 0.179	0.17, 0.179	0.179, 0.19	0.18, 0.194
Assisted Reproductive Technology	0.126	0.101	0.106	0.114	0.126	0.140	0.147	0.146
	0.117, 0.135	0.081, 0.121	0.09, 0.122	0.102, 0.126	0.115, 0.136	0.128, 0.152	0.132, 0.162	0.125, 0.166
Smoking	-0.114	-0.163	-0.131	-0.123	-0.103	-0.100	-0.111	-0.114
	-0.127, -0.101	-0.191, -0.135	-0.156, -0.106	-0.141, -0.105	-0.118, -0.087	-0.117, -0.083	-0.132, -0.09	-0.144, -0.084
Age (normalized)	0.019	0.013	0.012	0.016	0.019	0.022	0.022	0.025
	0.015, 0.022	0.005, 0.021	0.006, 0.018	0.011, 0.021	0.015, 0.023	0.018, 0.027	0.016, 0.028	0.017, 0.032
	-0.022	-0.020	-0.020	-0.023	-0.023	-0.022	-0.022	-0.023

Interaction term (GWG x BMI)	-0.025, -0.019	-0.027, -0.013	-0.025, -0.015	-0.027, -0.019	-0.026, -0.019	-0.026, -0.019	-0.028, -0.017	-0.029, -0.016

*All the p values for the association was less than 0.00001 except for maternal age at 5th quantile (p = 0.0021), maternal age at 10th quantile (p = 0.00017), and autoimmune disease at 95th quantile (p = 0.00056). BMI, body mass index; GWG, gestational weight gain

TABLE S4. Akaike's Information Criterion (AIC) scores of the multivariable quantile regression models

	AIC						
**quantile**	**5th**	**10th**	**25th**	**50th**	**75th**	**90th**	**95th**
Model 1	1304499	1204403	1075584	1016809	1057747	1164355	1249643
Model 2	1303686	1203508	1074631	1015607	1055963	1161941	1246486
Model 3	1277631	1181229	1060742	1008266	1051994	1160115	1246065
Model 4	1277598	1181234	1060711	1008061	1051329	1158849	1243949
Model 5	1277770	1181492	1061096	1008671	1052124	1159782	1244881
**Full model**	1277365	1181006	1060493	1007879	1051169	1158737	1243870

Portions of results are shown.

Model 1: covariates are maternal age, height, prepregnancy BMI, and smoking

Model 2: covariates are maternal age, height, prepregnancy BMI, smoking, DM, autoimmune disease, ART

Model 3: covariates are maternal age, height, prepregnancy BMI, smoking, HDP, autoimmune disease, ART

Model 4: covariates are maternal age, height, prepregnancy BMI, smoking, HDP, DM, ART

Model 5: covariates are maternal age, height, prepregnancy BMI, smoking, HDP, DM, autoimmune disease

Full model: covariates are maternal age, height, prepregnancy BMI, smoking, HDP, DM, autoimmune disease, ART

TABLE S5. Characteristics of a subsample of 859 women

	Mean [SD], Median (IQR) or n (%)
Prepregnancy BMI (kg/m^2)	20.2 (18.8, 21.9)
GWG (kg/ 40 weeks)	10.7 [3.3]
Height (cm)	158.9 [5.2]
Age (year)	32.6 [4.7]
Fetal sex (male)	460 (53.7)

BMI, body mass index; GWG, gestational weight gain

TABLE S6. Classification according to gestational weight gain adequacy at 40 weeks in the subsample population (n = 859)

		**GWG adequacy (at 40 weeks)**				
		**Inadequate**	**Adequate**	**Excessive**	**NA**	Total
**Underweight**	n	115	56	12	0	68
	(%)	(13.39%)	(6.52%)	(1.4%)	(0%)	(21.31%)
**Normal weight**	n	259	206	146	0	352
	(%)	(30.15%)	(23.98%)	(17%)	(0%)	(71.13%)
**Overweight**	n	19	7	23	0	30
	(%)	(2.21%)	(0.81%)	(2.68%)	(0%)	(5.7%)
**Obese**	n	0	0	10	6	16
	(%)	(0%)	(0%)	(1.16%)	(0.7%)	(1.86%)
**Total**	n	19	7	33	6	46
	(%)	(45.75%)	(31.31%)	(22.24%)	(0.7%)	(100%)

NA, not applicable. GWG, gestational weight gain

1. Sato N, Miyasaka N. Stratified analysis of the correlation between gestational weight gain and birth weight for gestational age: a retrospective single-center cohort study in Japan. BMC Pregnancy Childbirth. 2019;19(1):402.
2. **JSOG**. Guidelines for gestational weight gain 2021 [Available from: https://www.jsog.or.jp/news/pdf/20210616_shuuchi.pdf. (in Japanese)]
3. Morisaki N, Piedvache A, Morokuma S, Nakahara K, Ogawa M, Kato K, et al. Gestational weight gain growth charts adapted to Japanese pregnancies using a Bayesian approach in a longitudinal study: The Japan Environment and Children's Study. J Epidemiol. 2021.
4. Hulmán A, Witte DR, Kerényi Z, Madarász E, Tänczer T, Bosnyák Z, et al. Heterogeneous effect of gestational weight gain on birth weight: quantile regression analysis from a population-based screening. Ann Epidemiol. 2015;25(2):133-7, 7.e1.
